# Microstructure Evolution Study of the Field-Serviced Silicone Rubber Insulator via X-Ray Micro-CT Imaging

**DOI:** 10.3390/polym17223009

**Published:** 2025-11-12

**Authors:** Tiantian Chen, Fanglin Zha, Qian Luo, Lei Shi, Tao Wan, Bo Chen

**Affiliations:** 1 Key Laboratory of Advanced Civil Engineering Materials (the Ministry of Education), School of Materials Science and Engineering, Tongji University, Shanghai 201804, China; 2 State Grid Hunan Electric Power Company Limited Research Institute, Changsha 410007, China; 3 YonAudit Information Technology (Shanghai) Co., Ltd., Shanghai 200072, China

**Keywords:** composite silicone rubber insulator, insulator aging, X-ray computed tomography, three-dimensional structure, pores

## Abstract

Composite silicone rubber insulators are widely used in overhead transmission lines but undergo inevitable aging-induced performance degradation or failure. Understanding their aging process is crucial for optimizing manufacturing and on-site service, yet studies on microstructural changes—especially three-dimensional (3D) microstructural evolution during aging—remain scarce. This study employed X-ray micro-computed tomography (X-ray micro-CT) to characterize and compare the 3D microstructure of one fresh and one field-serviced composite silicone rubber insulator (which underwent 10 years of outdoor service). For the field-serviced insulator, key findings include the following: (1) pore number decreased (from 2200 to 1600), while pore size increased (equivalent diameter: from 3.5 ± 1.3 μm to 10.4 ± 7.2 μm); (2) porosity increased (from 0.04% to 0.69%); (3) new cracks formed (length: 100–900 μm); and (4) pore shape remained nearly unchanged relative to the fresh sample. Notably, the aging depth of the field-serviced insulator was less than 300 μm. These results clarify the 3D microstructural evolution of composite silicone rubber insulators during aging, providing a basis for improving their long-term reliability.

## 1. Introduction

Insulators serve as critical components in power transmission and transformation lines, with their operational condition being closely tied to the stability and safety of the power grid [1]. Their primary functions are to provide mechanical support and ensure electrical insulation. However, in outdoor environments, insulators are exposed to a range of adverse factors, including high temperatures, ultraviolet radiation, rain, and industrial pollutants such as dust and gases. These environmental stressors can result in various issues, such as self-explosion, physical damage, flashover, and the accumulation of contaminants, which compromise their performance and reliability [2]. For example, Xie’s research [3] has demonstrated that high temperature significantly enhances the reactivity of silicone rubber chains, thereby promoting the aging process. Hakimabadi et al. [4] demonstrated that UV radiation can alter the surface morphology of insulators, generating shallow cracks and causing the filler to be loosely bonded to the matrix, thus accelerating their aging process. Dong et al. [5] found that the local electric field distortion induced by raindrops causes insulator discharge. Fahmi et al. [6] show that the presence of salt pollutants can increase the partial discharge of insulators and thus shorten their service life.

Since the 1990s, composite insulators have been extensively utilized in transmission lines worldwide, gradually replacing ceramic and glass insulators. This is because the latter two types of insulators have significant mass and, thus, high transportation, installation, and maintenance costs [7]. Composite insulators are primarily composed of silicone rubber material, with polydimethylsiloxane (PDMS) serving as the matrix material. PDMS features a main chain structure consisting of Si-O-Si and side chains made of -CH_3_, which confer excellent hydrophobic properties. Additionally, the incorporation of fillers such as aluminum hydroxide (ATH; i.e., Al(OH)_3_) and silicon dioxide enhances the material’s mechanical strength and resistance to pollution flashover [8,9]. However, as the service life of composite insulators extends, they inevitably experience aging-related issues, including surface chalking, discoloration, brittleness, and compromised sealing. These aging phenomena can ultimately result in severe consequences, such as flashover incidents and power outages [7,10].

The microstructure of a material determines its mechanical and physical properties. Ning et al. [7] demonstrated that, in natural environments, the mechanical properties of silicone rubber deteriorate significantly when its surface morphology is damaged. Wang et al. [11] indicated that when silicone rubber insulators exhibit surface discoloration—i.e., when their surface structure is damaged—both their mechanical properties and hydrophobicity decrease. Ullah’s research [12] showed that the hydrophobic and electrical properties of insulators degrade when their surface structure is damaged. Therefore, it is crucial to analyze the microstructure of insulators in as much detail as possible. Traditional two-dimensional (2D) characterization methods have inherent limitations, particularly their inability to provide information about the spatial distribution of key features. Three-dimensional (3D) imaging techniques overcome these limitations by enabling the identification of spatial locations of cracks, pores, impurities, and different phases within the materials [13].

X-ray computed tomography (X-ray CT) is a 3D imaging technique capable of achieving micrometer or even nanometer resolution in the structural characterization of materials [13]. Initially developed in the 1970s by Godfrey Hounsfield for medical diagnostics, X-ray CT has undergone significant advancements in resolution in recent decades [14]. Modern X-ray CT systems, such as X-ray micro-computed tomography (X-ray micro-CT) [15], can achieve resolutions ranging from 0.3 mm to 0.5 μm or even higher [16]. While X-ray CT was originally confined to medical applications, the enhanced resolution and versatility of X-ray micro-CT have enabled its adoption across a wide range of fields, including food science, medical science, earth science, life science, geological science, materials science, nanotechnology, additive manufacturing, and tissue engineering [13,17,18,19,20]. Beyond its qualitative diagnostic capabilities, with the help of image analysis software, X-ray micro-CT also facilitates quantitative analyses, such as determining shapes, sizes, connectivity, orientation, spatial distribution, and the volume fraction of features such as target phases or pores in the samples [21,22,23,24]. In existing studies, techniques such as Attenuated Total Reflection Fourier-Transform Infrared Spectroscopy (ATR-FTIR), Laser-Induced Breakdown Spectroscopy (LIBS), and Proton Transfer Reaction Spectroscopy (PTR) have been utilized to confirm that the aging of composite silicone rubber insulators leads to surface enrichment. Wang et al. [25] utilized Laser-Induced Breakdown Spectroscopy to determine the aging depth of insulators, which was 200 μm. Jiang et al. [26] employed the photothermal radiometry method to measure the aging depth of insulators that had been in service for 10 years, which was 62 μm. Additionally, Vásárhelyi et al. [13] used the microscopic infrared method to measure the aging depth of insulators that served for 10 years, which was 141 μm.

X-ray micro-CT can obtain 3D spatial structural images of materials with micrometer resolution. Compared with other conventional methods, it is a non-destructive, high-resolution approach for structural characterization and is starting to be used in research on the microstructure of insulators [13,15,27]. Kim et al. [28] and Jeon et al. [29] utilized X-ray micro-CT to investigate ceramic insulators, but few studies have explored its application in composite insulators. Nevertheless, X-ray micro-CT has been extensively employed to analyze various composite materials [30,31,32]. J.R. Innes and colleagues [33] used this technique to measure the pores formed during the fused filament fabrication (FFF) process, which revealed the structural characteristics of the samples. Similarly, Enzo Moretto et al. [34] demonstrated that dendritic particles enhance the mechanical reinforcement properties of rubber-based nanocomposites through mechanical testing and advanced imaging techniques, including X-ray micro-CT. Kuang et al. [35] employed X-ray micro-CT to investigate the origin and propagation mechanisms of severe damage in EPDM O-rings exposed to high-pressure hydrogen environments. Additionally, the 3D microstructure and compositional heterogeneity of multi-walled carbon nanotube/magnetite/polyurethane composites were analyzed using this technique [36]. Wang et al. [37] applied X-ray micro-CT to perform layer-by-layer scanning of U-shaped glass fiber-reinforced polypropylene (GFRPP) rails, enabling the analysis of shear angle variations across different layers and regions within the same layer. These studies underscore the versatility and precision of X-ray micro-CT in advancing the understanding of the structures of composite materials and their properties.

This work investigates the effects of long-term real outdoor field service on the microstructure of composite silicone rubber insulators by analyzing the 3D spatial structural differences between two samples: a fresh composite silicone rubber insulator and a sample extracted from the umbrella skirt portion of a composite silicone rubber insulator after 10 years of outdoor on-site operation. The analysis is conducted using X-ray micro-CT measurements, which provide detailed insights into the microstructural changes induced by environmental exposure over time. This approach aims to elucidate the impact of long-term outdoor conditions on the structural integrity and performance of silicone rubber insulators. This study proposes the following hypothesis: long-term outdoor service exposure increases the porosity and crack density in the near-surface layer of composite silicone rubber insulators while preserving the anisotropy of pore shape. Comparison of the 3D microstructures of fresh and field-serviced insulators via X-ray micro-CT can verify this hypothesis and quantify relevant change patterns.

## 2. Materials and Methods

### 2.1. Sample Preparation

Two sets of samples were prepared for this study. The first set of samples, referred to as the “fresh insulator,” consisted of newly manufactured composite silicone rubber materials. The second set, designated as the “field-serviced insulator”, comprised composite silicone rubber insulator umbrella skirt components that had experienced 10 years of outdoor operation in Hunan Province, China. Both sets of samples were sourced from the same manufacturer to achieve as much consistency in material composition and production processes as possible.

### 2.2. X-Ray Micro-CT Measurements

X-ray micro-CT is a non-destructive imaging technique that helps obtain 3D structural information [38]. The equipment used in this study was an X-ray micro-CT instrument (Zeiss Xradia 520 Versa, Zeiss, Oberkochen, Germany) operated at a voltage of 80 kV with a power of 7 W. During the tomographic scanning process, the samples were rotated 180°. A total of 2201 projections were acquired for each 3D tomographic scan with an exposure time of 3 s for each projection. The pixel size of the obtained images was 3.5 μm for both samples, and the minimum distinguishable pore/crack width was approximately 2–3 voxels. With the above-mentioned setup, each scan took approximately 2 h. The source–sample distance was −8.59, and the sample–detector distance was 30.51; the source filter was LE1. For reconstruction, the algorithm was Auto CB, the voxel pitch was associated with the 4X objective and binning 2 (corresponding to a resolution related to 3.5 μm, as indicated by the recipe point name), beam-hardening and ring-artifact corrections were implicitly addressed by the Auto CB reconstruction and multi-reference collection, and phase retrieval was not employed. The samples were vertically fixed on the sample stage with hot-melt adhesive—specifically, the surface normal of the samples was kept perpendicular to the rotation axis of the CT scan.

The software Avizo 2021.1 was employed for post-processing and quantitative analysis of the image data. First, pre-processing was carried out: parameters such as the brightness and contrast of the reconstructed tomogram slices were adjusted to maximize the grayscale value differences among different structures to achieve the optimal state for grayscale segmentation, and anisotropic diffusion filtering was applied for noise reduction. After that, the Interactive Thresholding function in Avizo was utilized to separate the sample from the surrounding air based on the grayscale differences, as well as different regions within the sample, including the matrix, fillers, and pores. Global thresholding was adopted, and the watershed algorithm was not used. Size-based cleanup was performed via a minimum volume threshold, with connectivity defined as 26-neighborhood. Subsequently, the 3D reconstructed data of the 2 samples were cut into the same size (800 μm × 800 μm × 900 μm), and the pores and cracks within the samples were extracted. After that, the Label Analysis function was employed to obtain quantitative data such as the number and size of the pores and cracks. The overall porosity of the samples was defined as the ratio of the total pore volume to the total volume of the sample, while the fractal dimension was calculated using the Fractal Dimension algorithm to characterize the surface roughness of the samples.

## 3. Results

**Figure 1:**
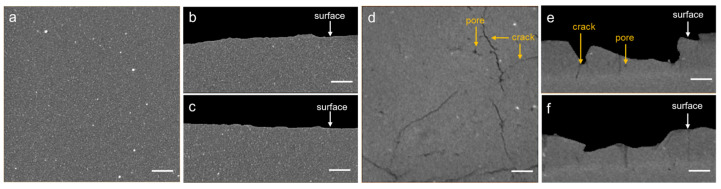
The reconstructed tomogram slices of the fresh insulator and the field-serviced insulator. Top view (**a**) and side views (**b**,**c**) of the fresh insulator. Top view (**d**) and side views (**e**,**f**) of the field-serviced insulator. Pores and cracks (pointed by orange arrows) could be easily found in the field-serviced insulator. The silicone rubber matrix is shown in gray, inorganic fillers in white, and pores/cracks in black. The scale bars are 100 μm.

Figure 1 presents the reconstructed tomogram slices of both the fresh insulator and the field-serviced insulator. In these images, the gray regions correspond to the matrix component—i.e., PDMS—where the white areas represent the filler particles and the black regions indicate the pores or cracks. In the fresh insulator (Figure 1a–c), only the matrix and filler particles are visible, which suggests a uniform and intact structure. In contrast, the field-serviced insulator (Figure 1d–f) does not only have matrix and filler particles, but also pores and cracks. This means that obvious structural changes occurred after field service.

**Figure 2:**
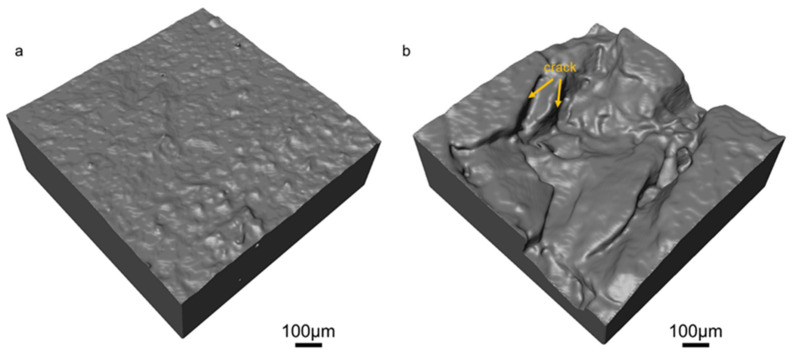
The surface of (**a**) the fresh insulator and (**b**) the field-serviced insulator. Cracks (pointed by orange arrows) could be easily found on the surface of the latter. Gaussian smoothing was applied to the surface to eliminate image noise.

Figure 2 presents the surface structures of both the fresh insulator and the field-serviced insulator, revealing notable differences in their morphology. The surface of the fresh insulator appears relatively flat and smooth, with only a few small granular structures visible. In contrast, the field-serviced insulator exhibits a much rougher surface with prominent pits and cracks. To quantify these structural differences, the fractal dimensions of the two samples were calculated using the Avizo software. The fractal dimension is a measure of the complexity and roughness of a fractal object, where a higher fractal dimension indicates greater surface complexity and roughness [39]. Liu et al. have proven that an increase in the fractal dimension signifies more severe damage to concrete samples and a decline in their fatigue resistance [40]. The fractal dimension was 1.47 for the fresh insulator and 1.84 for the field-serviced insulator (as presented in Table 1). The increase in the fractal dimension indicates a rougher surface, which would lead to deterioration of the performance of the insulator while serving in outdoor environments, making the aged insulator more susceptible to damage. The larger fractal dimension of the field-serviced insulator corroborates the visual observations shown in Figure 2.

**Figure 3:**
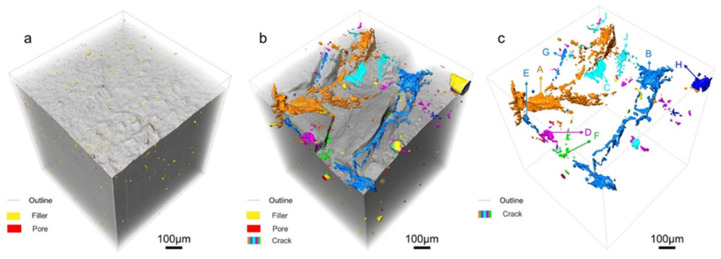
The 3D spatial structure of (**a**) the fresh insulator and (**b**) the field-serviced insulator (both presented as full-volume renderings). (**c**) The spatial distribution of cracks in the field-serviced insulator. The silicone rubber matrix is displayed in gray, inorganic fillers in yellow, pores in red, and cracks in multiple colors.

Figure 3 illustrates the 3D spatial structure of both the fresh and field-serviced insulators. In these images, the gray lines show the edges of the samples, the filler particles are yellow, the pores are red, the cracks are shown in other colors, and the gray part presents the matrix PDMS. Figure 3a shows that besides filler particles, the fresh insulator contains fine pores as well, which are mainly due to incomplete venting and water vapor evaporation during the vulcanization phase [41,42]. Figure 3b shows that both pores and cracks appeared in the field-serviced sample. This result is consistent with the findings presented in Figure 1. During service, the samples are subjected to environmental factors such as corona discharge, ultraviolet irradiation, and wind, sand, and chemical erosion, which results in the detachment of filler particles and the development of inherent pores in the material, ultimately leading to the generation of pores and cracks [41,43,44]. Fillers, as inorganic rigid components, do not physically grow during service. Therefore, the apparent increase in filler size is due to their good dispersibility in the fresh sample, allowing for clear identification of individual particles during segmentation. However, in the field-serviced sample, the silicone rubber matrix degrades due to aging, causing some filler particles to lose matrix encapsulation and undergo slight agglomeration. The agglomerated bright regions were misclassified as “single large-sized fillers” in the segmentation process, leading to an overestimated average diameter.

**Figure 4:**
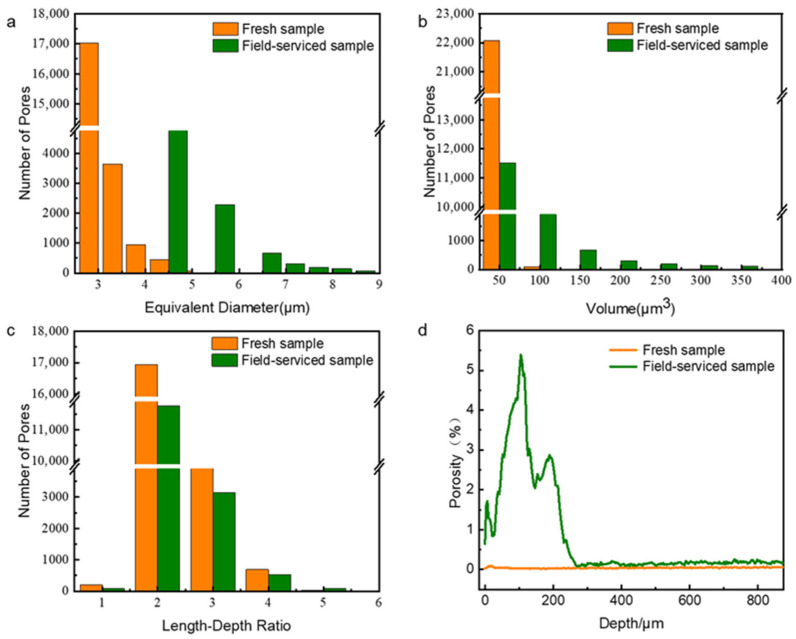
Comparison of the pore structure of the fresh and field-serviced insulators: (**a**) Equivalent diameter, (**b**) volume of pores, (**c**) length–depth ratio, and (**d**) porosity.

Figure 3c illustrates the cracks only in the field-serviced insulator, with detailed dimensions provided in Table 2. Length is defined as the distance between the two endpoints of a crack, width refers to the average distance between the two side edges of the crack in the direction perpendicular to its extension, and depth represents the distance that the crack extends into the interior from the surface of the sample along the direction perpendicular to this surface. Sphericity represents the compactness of the crack, where the value for a perfect sphere is 1. The calculation formula for sphericity is shown in Equation (1), where ϕ is sphericity, V is the volume of the crack, and A is the total surface area of the crack. The larger the sphericity, the less compact the crack. The average length of all the cracks in the field-serviced insulator is 122.7 μm, and the average width and depth are 43.3 μm and 35.7 μm, respectively. Figure 3 and Figure 4 show that the cracks predominantly originated and developed on the surface of the sample with a depth of about 200 μm after 10 years of on-site service. Furthermore, the dimensional data presented in Table 2 reveal that the extent of crack propagation along the surface direction significantly exceeds that of cracks growth in the depth direction of the insulator. Moreover, the sphericity of all the cracks is greater than 20, which indicates that the cracks have a strong tendency to propagate. They extend in multiple directions in the 3D space and develop in a relatively loose and open manner, rather than being confined to a relatively compact area.

(1) $$\phi =\frac{\pi^{\frac{1}{3}}{\left(6V\right)}^{\frac{2}{3}}}{A}$$

Figure 4 presents the results of the quantitative analysis of the structural composition of both the fresh insulator and the field-serviced insulator. As illustrated in Figure 4a, the fresh insulator contains more pores compared with the field-serviced insulator; however, the average pore size in the fresh insulator is smaller. This can be attributed to the aging process of composite silicone rubber insulators during service. Over time, the small pores that initially formed during the production process gradually expand, and pore combination occurs, leading to an increase in pore size and a decrease in pore number. Specifically, the number of pores in the fresh insulator is approximately 22,000, and the equivalent diameter ranges from 3 to 4 μm. (The equivalent diameter is calculated by measuring the 3D volume of the pore and then equating it to a standard sphere with the same volume. The diameter of this standard sphere is defined as the equivalent diameter.) In contrast, for the field-serviced insulator, the number of pores is about 16,000, and the equivalent diameter increases to a range of 5 to 7 μm. This means that the number of pores decreases by approximately 27%, while the equivalent diameter increases by about 70%.

The distribution of the pore volumes is shown in Figure 4b. In the fresh insulator, the volume of almost all the pores is less than 50 μm^3^, while in the field-serviced insulator, the pore volume significantly expands to a range of 50 to 150 μm^3^. Additionally, Figure 4c illustrates that in both the fresh and field-serviced insulators, the length–depth ratio of the pores exceeds 1, which indicates that pore expansion occurs more prominently in the surface direction than in the depth direction. This is because that the surface of the sample is directly exposed to the environment and is subjected to more complex forces, resulting in more significant expansion of pores or cracks along the surface direction [45,46].

Table 1 shows that the fresh insulator has a porosity of 0.04%, whereas that of the field-serviced insulator is significantly higher at 0.69%, which indicates that the latter has a weakened structural composition. Further insights into the porosity distribution from the surface to the inner part of the samples (from X-ray micro-CT measurements) are shown in Figure 4d. Slice 0 (in the reconstructed tomographic 3D images) corresponds to the surface of the samples and, the greater the slice number, the deeper the sample is sliced. The porosity per slice in the fresh insulator remains consistently close to 0, reflecting its uniform and compact structure. In contrast, the field-serviced insulator exhibits fluctuations in porosity values across different depths, attributed to the non-uniform distribution of its pore structure. Notably, the porosity of the field-serviced insulator is higher than that of the fresh insulator from 0 to 262.5 μm (the 75th slice of the 3D image). The porosity of both samples is almost constant, which indicates that the aging depth of the field-serviced insulator is about 262.5 μm, remaining less than 300 μm even after 10 years of on-site service. This is consistent with the results reported by Wang et al. [25], Jiang et al. [26], and Vásárhelyi et al. [13]. It should be noted that the differences in these results of the insulators’ aging depth are due to factors such as the number of served years, the operation environment, and the insulators’ composition and manufacturing process.

## 4. Discussion

Hunan Province, located in the south–central region of China, experiences high temperatures and heavy rainfall during the summer. The annual average temperature in this region is 17.7 °C, and the maximum temperature can reach 42 °C. The annual average sunshine duration is 1422.6 h, and the annual average precipitation is 1435.6 mm. These environmental conditions subject insulators in service to prolonged high-temperature exposure and significant impacts from rainfall, which accelerate the aging process of the composite silicone rubber umbrella skirts used in the insulators. The primary material used in these umbrella skirts is PDMS, a polymer composed of a siloxane main chain and methyl side chains. The chemical bonds within PDMS exhibit varying bond energies: the silicon—oxygen bond has a bonding energy of approximately 447 kJ/mol, that of the silicon—carbon bond is about 318 kJ/mol, and that of the carbon—hydrogen bond is around 413 kJ/mol [47]. However, ultraviolet (UV) rays from solar irradiation possess sufficient energy to break these bonds, leading to the formation of Si• and Si-CH_2_•. These radicals subsequently react with moisture in the air, producing hydrophilic groups such as Si-OH and Si-COOH [48,49]. As a result, the hydrophobicity of the sample surface diminishes, allowing moisture to adhere to the surface and penetrate the material through pre-existing pores and cracks formed during aging [50].

This moisture infiltration exacerbates the aging process by extending it into the interior of the material. While moisture penetrates into the material, oxidation and hydrolysis reactions occur in PDMS. The Si-O-Si main chain breaks and combines with -OH and then undergoes a re-crosslinking reaction. This process disrupts the 3D network structure of the material, leading to the deterioration of its mechanical properties [51,52,53].

(2) $${Al\left(OH\right)}_{3}+{3H}^{+}⇌{Al}^{3+}+3H_{2}O$$

(3) $${Al\left(OH\right)}_{3}+H_{2}O+⇌{{Al\left(OH\right)}_{4}}^{-}+H^{+}$$

In addition to containing PDMS, the samples also include fillers such as ATH and silica. ATH’s amphoteric nature makes it susceptible to chemical reactions in weakly acidic environments, such as rainwater, which typically has a pH value of 5.6–7. Under these conditions, ATH reacts to form Al^3+^ and Al(OH)^4−^, as illustrated in Equations (2) and (3), and these reaction products are subsequently washed away by rain. This process reduces the concentration of filler on the sample’s surface, leading to the formation of pores [44,53]. Furthermore, the weak bonds between PDMS and fillers like ATH exacerbate this issue, as prolonged exposure to solar radiation can cause photodegradation of the PDMS matrix, further contributing to the loss of fillers [54]. This explains why the porosity of the field-serviced insulator increased and why the number of pores in the field-serviced insulator decreases while their sizes increase.

These pores provide initiation sites for the formation of cracks and influence the crack propagation paths. Moisture adhering to the surface of the material penetrates into the interior of the sample through the pores, which generates a wedging effect at the crack tips. Since water expands in volume when it freezes, this expansion force will cause stress concentration at the crack tips, thereby promoting the further propagation of cracks [55,56]. Additionally, the stress distribution within the material is uneven, and the phenomenon of stress concentration is more pronounced at defects and cracks. Under the continuous action of environmental stress, the material in these stress-concentrated areas gradually undergoes yielding and fracture. As a result, the cracks continuously propagate towards the surrounding areas, accelerating the aging and damage of the material [42]. Therefore, during the service life of the composite silicone rubber samples, moisture invades from the surface to the interior through the existing pore structure, damaging the 3D structure of PDMS. Coupled with the effect of rain scouring, new pores are continuously generated and merge with existing pores, which leads to an increase in porosity and an enlargement in pore size. Subsequently, cracks are formed, causing the deterioration of their mechanical and electrical properties.

## 5. Conclusions

X-ray micro-CT is an effective and non-destructive technique for measuring microstructural changes in composite silicone rubber insulators. In this study, X-ray micro-CT was employed to measure the number, size, shape, overall porosity, and spatial structure of both fresh and field-serviced insulators. We found significant differences between the two samples. The fresh insulator has small pores (average diameter of 2.77 μm) but a higher number of pores, primarily attributed to the production process. In contrast, the number of pores in the field-serviced insulator was reduced by approximately 6000, about 29% that of the fresh insulator with the same volume; furthermore, the pore size increased to 4.97 μm, and large cracks were formed, which contributed to a rougher surface. Additionally, the overall porosity of the field-serviced insulator increased to 0.69% (0.04% for the fresh insulator). However, the shape of the pores in the field-serviced insulator remained largely consistent with that observed in the fresh insulator. Importantly, even after 10 years of outdoor field service, the aging depth of the composite silicone rubber insulators was about 262.5 μm, less than 300 μm, indicating that thicker insulators may provide higher safety during on-site service. Based on these results, X-ray micro-CT is demonstrated an effective detection method for microstructural changes in composite silicone rubber insulators, which can be applied to determine the aging degree of insulators and provide a basis for the maintenance and replacement of field-serviced insulators.

## Figures and Tables

**Table 1:** Detailed information of each structure in the fresh insulator and the field-serviced insulator.

	Fresh Insulator	Field-Serviced Insulator
Fractal dimension of surface	1.5	1.8
Porosity (%)	0.04	0.69
Average diameter of fillers (μm)	3.5 ± 1.3	10.4 ± 7.2
Average diameter of pores (μm)	2.8 ± 0.4	5.0 ± 1.5
Average length of cracks (μm)	-	118.2

**Table 2:** Crack sizes in the field-serviced insulator.

	Length (μm)	Width (μm)	Depth (μm)	Volume (×10^4^ μm^3^)	Sphericity
A *	842.7	243.4	211.0	122	566.3
B *	838.3	204.0	182.8	89.1	423.7
C	181.3	53.6	47.1	10.3	36.9
D	150.3	76.5	42.8	7.04	32.7
E *	140.2	43.0	18.6	2.34	27.3
F *	170.6	41.5	32.8	4.14	34.7
G	235.5	44.9	33.3	3.28	31.9
H *	132.5	88.2	83.6	13.8	39.5
I	129.5	51.6	39.8	1.88	21.7
J	181.5	95.4	95.2	7.34	74.0

* Denotes a boundary-touching crack.

## Data Availability

The original contributions presented in this study are included in the article. Further inquiries can be directed to the corresponding authors.
